# Genome-Wide Identification and Characterization of Gibberellic Acid-Stimulated Arabidopsis Gene Family in Pineapple (*Ananas comosus*)

**DOI:** 10.3390/ijms242317063

**Published:** 2023-12-02

**Authors:** Mingzhe Yang, Chaoyang Liu, Wei Zhang, Jing Wu, Ziqin Zhong, Wen Yi, Hui Liu, Yan Leng, Weisheng Sun, Aiping Luan, Yehua He

**Affiliations:** 1 Key Laboratory of Biology and Germplasm Enhancement of Horticultural Crops in South China, Ministry of Agriculture and Rural Areas, College of Horticulture, South China Agricultural University, Guangzhou 510642, China; 20213137174@stu.scau.edu.cn (M.Y.); liuchaoyang@scau.edu.cn (C.L.); vivi950212@scau.edu.cn (W.Z.); 20211016009wj@stu.scau.edu.cn (J.W.); ziqinzhong@stu.sicau.edu.cn (Z.Z.); 20wenyi@stu.scau.edu.cn (W.Y.); huihuitwins@stu.scau.edu.cn (H.L.); 202131317122@stu.scau.edu.cn (Y.L.); 2 South Subtropical Crop Research Institute, Chinese Academy of Tropical Agricultural Sciences, Zhanjiang 524091, China; sunweisheng1234@sina.com; 3 Tropical Crops Genetic Resources Institute, Chinese Academy of Tropical Agricultural Sciences, Haikou 571101, China

**Keywords:** gibberellic acid-stimulated Arabidopsis, pineapple, plant growth, expression patterns

## Abstract

The gibberellic acid-stimulated Arabidopsis (GASA) gene family plays a crucial role in growth, development, and stress response, and it is specific to plants. This gene family has been extensively studied in various plant species, and its functional role in pineapple has yet to be characterized. In this study, 15 *AcGASA* genes were identified in pineapple through a genome-wide scan and categorized into three major branches based on a phylogenetic tree. All AcGASA proteins share a common structural domain with 12 cysteine residues, but they exhibit slight variations in their physicochemical properties and motif composition. Predictions regarding subcellular localization suggest that AcGASA proteins are present in the cell membrane, Golgi apparatus, nucleus, and cell wall. An analysis of gene synteny indicated that both tandem and segmental repeats have a significant impact on the expansion of the *AcGASA* gene family. Our findings demonstrate the differing regulatory effects of these hormones (GA, NAA, IAA, MeJA, and ABA) on the *AcGASA* genes. We analyzed the expression profiles of *GASA* genes in different pineapple tissue parts, and the results indicated that *AcGASA* genes exhibit diverse expression patterns during the development of different plant tissues, particularly in the regulation of floral organ development. This study provides a comprehensive understanding of *GASA* family genes in pineapple. It serves as a valuable reference for future studies on the functional characterization of *GASA* genes in other perennial herbaceous plants.

## 1. Introduction

The GASA gene family is now widely observed in monocotyledonous and dicotyledonous plants [1]. These genes, belonging to the GASA family, are characterized by their cysteine-rich peptides and possess a conserved structural domain primarily comprising 12 cysteines at the C-terminus [2,3]. Over the past few decades, *GASA* family genes have been identified in numerous plant species, including *Arabidopsis thaliana* [3], tobacco (*Nicotiana tabacum*) [4], rice (*Oryza sativa*) [5], Grapevine (*Vitis vinifera* L.) [6], potato (*Solanum tuberosum*) [7], and poplar (*Populus trichocarpa*) [8]. However, it has yet to be detected in charophytes or bryophytes [9,10].

GASA proteins have been demonstrated to play a role in regulating various plant growth and developmental processes, including fruit maturation and development [11], cell division [12], organ development [13], lateral root development [14], stem elongation [15], and seed germination [6,16]. In Arabidopsis, AtGASA4 promotes the flower development process, while the overexpression of *AtGASA5* leads to delayed flowering due to the downregulation of LFY (LEAFY) and FT (FLOWERING LOCUS T) expression and upregulation of FLC (FLOWERING LOCUS C) expression [15]. In the case of petunia, GASA proteins contribute to shoot elongation [17].

Additionally, the *GASA* gene exhibits tissue-specific expression patterns. For example, the *GASA* gene is highly expressed during both the ripening and flowering stages of tomato fruit [18]. In rice, the *GASR9* gene exhibits an elevated expression in the panicles [19], while in Arabidopsis, *GASA1* and *GASA2* are prominently expressed in floral buds [20].

Interestingly, *GASA* family genes play a vital role in responding to stress and hormonal responses. Numerous studies have confirmed their resistance to salt, drought, and high temperatures [21,22]. For instance, the overexpression of *FsGASA4* increases tolerance to salt, oxidative stress, and heat in beechnut (*Fagus sylvatica*) and transgenic Arabidopsis [23]. Similarly, the overexpression of *GsGASA1* in soybeans inhibits low-temperature root growth [24]. Moreover, GASA proteins play a crucial role in enhancing plant resistance and its antimicrobial properties [25,26,27]. For instance, following infection with the citrus tristeza virus, citrus leaves exhibit a substantial upregulation of *CcGASA4* expression [28]. In Arabidopsis and soybean, the overexpression of the *GASA* family gene *GmSN1* enhances resistance to viral infections [29]. Notably, the antifungal properties of *GASA* members have been documented in Arabidopsis [30], tomato [31], and alfalfa [9]. 

Furthermore, *GASA* genes are influenced by hormones such as gibberellin (GA), abscisic acid (ABA), naphthalene acetic acid (NAA), and salicylic acid (SA). Most *GASA* genes are involved in the regulation of the gibberellin pathway. For instance, in Arabidopsis, *AtGASA4*, *AtGASA6*, *AtGASA7*, *AtGASA8*, and *AtGASA13* are induced by GA [30,32]. It is worth noting that *AtGASA4* is down-regulated in GA-treated cotyledons and leaves but up-regulated in most meristematic tissue regions [3]. Additionally, it has been observed that growth hormones, such as GA, auxin, and cytokinin, can stimulate the expression of the *AtGASA4* and *AtGASA6* genes in Arabidopsis. Conversely, ABA, SA, and MeJA have been found to exert a repressive effect on the expression of these genes [33]. In Arabidopsis, *AtGASA6* acts as a mediator in the signaling pathways of gibberellin, abscisic acid, and glucose during seed germination [34]. In rice, the expression of *OsGASR1* and *OsGASR2* is induced by GA [5]. *OsGASR1* functions as a mediator between brassinosteroid (BR) and GA signaling pathways [35]. Similarly, in Glycine max, GA has been found to up-regulate *GmGASA32*, which promotes plant height by interacting with GmCDC25, which is a cell cycle-associated protein [36]. ABA has been demonstrated to exert both promotive and suppressive effects on the expression of *GASA* genes [27,30,37]. In potatoes, the application of ABA induced the expression of *StSN2* [27] while repressing the expression of *Snakin-3* [7]. In tomatoes, the upregulation of *GAST1* transcript levels following GA treatment was partially inhibited by ABA [38]. Furthermore, GA and ABA have been found to effectively enhance the expression of *MdGASA* during the flowering stage of apple (Malus domestica) [39]. Additionally, MeJA treatment significantly upregulated the transcription of *HbGASA7-1*, *14*, and *16* in Hevea brasiliensis [40].

Pineapple is one of the world’s top cultivated tropical fruits and is known as the queen of fruits. It holds considerable medical and industrial significance due to its fiber content and nutritional value [41,42,43]. Pineapple growth and development are susceptible to adverse conditions such as high temperatures and fluctuation in hormone levels, which can substantially impact both yield and quality. To preserve the commercial value of pineapples, it is crucial to explore the mechanisms governing plant growth, development, and stress response. The *GASA* gene family plays a crucial role in plant growth and development, but its presence and characteristics in pineapple have remained unexplored. In this study, we employed bioinformatic techniques to identify 15 *GASA* family genes in pineapple. We analyzed their gene structures, phylogenetic relationships, protein chemical properties, motif compositions, and genomic collinearity. Additionally, we investigated the potential functions of *AcGASA* genes in pineapple by analyzing pineapple transcriptome data and examining how the expression analysis of *GASA* genes responds to hormone treatments. The findings of this experiment provide a basis for further studies on the identification of *GASA* genes in pineapple and other fruit trees.

## 2. Results

### 2.1. Physicochemical Properties of the AcGASA Gene

We identified 15 *AcGASA* family genes in pineapple using BlastP and HMMER, and we assigned them the names *AcGASA1-15* based on their order in the chromosome (Table S1). These genes exhibited a range of characteristics (Table 1), with amino acid counts varying from 63 (*AcGASA3/6*) to 155 (*AcGASA8*), molecular weights (MWs) ranging from 6.67 (*AcGASA6*) to 17.39 (*AcGASA8*) KDa, isoelectric points (pIs) ranging from 8.48 (*AcGASA2*) to 9.25 (*AcGASA8*), and their aliphatic index ranging from 17.14 (*AcGASA3*) to 74.00 (*AcGASA9*). With the exception of *AcGASA2*, *AcGASA5*, and *AcGASA7*, the hydrophilicity scores (GRAVY) for the other *AcGASA* genes were negative, indicating their hydrophilic nature. Subcellular localization predictions for all *AcGASA* genes showed that *AcGASA* genes were localized to different locations, including the cell membrane, Golgi, nucleus, and cell wall. 

### 2.2. Characterization of the AcGASA Genes

**Figure 1:**
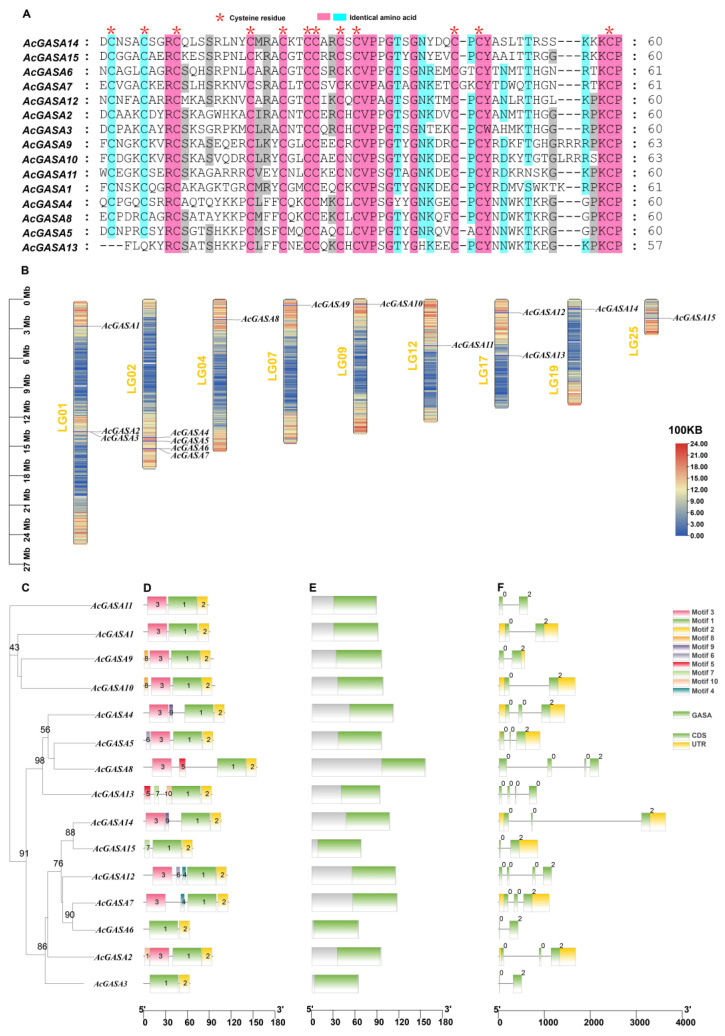
Conserved sequence, chromosomal distribution, gene structure, and gene motifs of the pineapple *AcGASA* gene. (**A**) A comparison of the AcGASA protein’s GASA structural domains with conserved cysteines is represented by red asterisks. (**B**) The chromosomal distribution of *AcGASA* genes with gene density is indicated from blue (low) to red (high). (**C**) The phylogenetic tree of *AcGASA* genes. (**D**) AcGASA protein-conserved motifs. (**E**) The *AcGASA* structural domain. (**F**) The *AcGASA* gene structure. Exons are represented by green, untranslated regions are substituted with yellow boxes, and black lines indicate introns.

The GASA proteins exhibit conserved C-terminal structural domains, each of which encompasses 12 conserved cysteines [3]. In pineapple, we observed a similar pattern to tobacco, with 12 conserved cysteines in all pineapple GASA proteins, except for AcGASA13 (Figure 1A). This divergence could be attributed to amino acid insertions and deletions [4].

The chromosomal positions of the *AcGASA* gene in the pineapple are shown in Figure 1B. We identified tandem repeats for *AcGASA2*, *AcGASA3*, *AcGASA6*, and *AcGASA7* (Figure 1B). Additionally, the occurrence of more than two *GASA* genes on a single chromosome was primarily located on LG1\2\17, with most *AcGASA* genes clustering in regions of a high chromosome density (Figure 1B).

We identified a limited number of conserved motifs in the *AcGASA* gene, ranging from 2 to 5, which contrasts with our initial expectation of 10 conserved motifs (Figure 1D and Figure S1). Motifs 1 and 2 were present in all GASA proteins, with motif 1 being a component of the GASA structural domain, while motif 3 was found in most GASA proteins. The number of exons varied from two to four, and only seven genes possessed complete 5′ or 3′ UTR regions (Figure 1F).

### 2.3. Developmental Relationships of Different Species of the GASA Family

**Figure 2:**
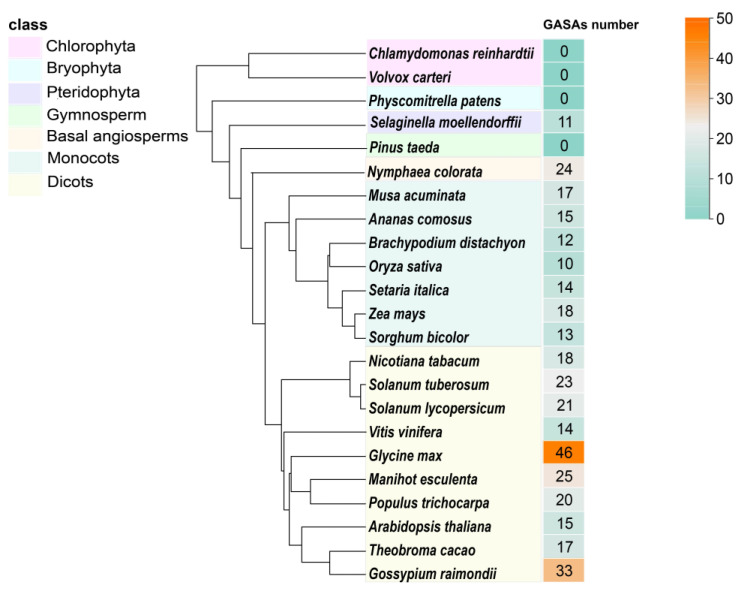
An evolutionary tree and the number of gibberellic acid-stimulated Arabidopsis (GASA) genes for 23 species. Various colors are employed to represent different classifications, including *Chlorophyta*, *Bryophyta*, *Pteridophyta*, *Gymnosperms*, *Basal Angiosperms*, *Monocots*, and *Dicots*. The GASA number column provides the number of *GASA* genes in each species. The light blue to yellow column shows the amount of *GASA* genes in each species, from low to high.

In our initial analysis, we analyzed the evolutionary history of *GASA* genes across 23 different species. We identified 366 *GASA* genes from 19 species, with some exceptions including *Pteridophyta*, *Basal angiosperms*, *Monocots*, and *Dicots* (Figure 2). Notably, GASA proteins are prevalent in higher plants but absent in *Gymnosperms* (*Pinus taeda*), as well as *Chlorophyta* (*Chlamydomonas reinhardtii* and *Volvox carteri*) and *Bryophyta* (*Physcomitrella patens*). These *GASA* genes exhibited varying numbers across different species, typically falling within the range of 10 to 20. Some species, like *Glycine max* (46) and *Gossypium raimondii* (33), featured more GASA proteins. There were 11 GASA proteins in *Pteridophyta* (*Selaginella moellendorffii*). Among the monocots, the number of *GASA* genes ranged from 10 to 18, while in Dicots, this range extended from 14 to 46. In conclusion, *Dicots* exhibited a higher number of *GASA* genes compared to *monocots*.

### 2.4. Phylogenetic Analysis of the GASA Family Genes

**Figure 3:**
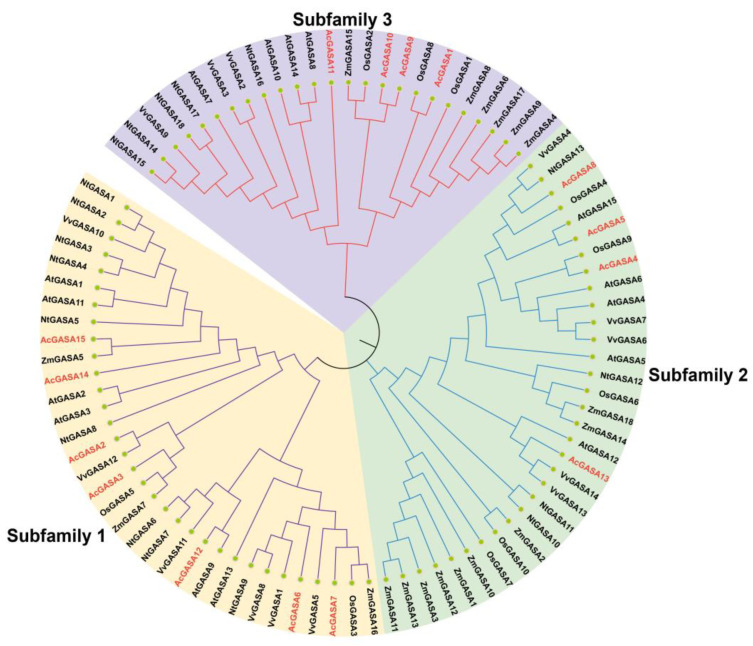
The phylogenetic tree of *GASA* genes from pineapple, *Arabidopsis thaliana*, maize, tobacco, grape, and rice. These GASA proteins across the six species were used to create neighbor-joining (NJ) trees supported by 1000 bootstrap replicates. The *GASA* genes are categorized into three distinct groups (Subfamily 1, Subfamily 2, and Subfamily 3), and the pineapple *GASA* genes are highlighted in red.

To explore the evolutionary relationships among pineapple (*A. comosus*), Arabidopsis (*A. thaliana*), tobacco (*N. tabacum*), rice (*O. sativa*), grape (*V. vinifera*), and maize (*Z. mays*), we constructed a phylogenetic tree that encompassed a total of 90 *GASA* genes (Table S2). This evolutionary tree was categorized into three branches, as previously described [4]. Our analysis revealed that Subfamily 1 contained 33 genes, Subfamily 2 had 32 genes, and Subfamily 3 encompassed 25 genes (Figure 3). Within the pineapple species, we identified seven *GASA* genes (*AcGASA2*, *AcGASA3*, *AcGASA6*, *AcGASA7*, *AcGASA12*, *AcGASA14*, and *AcGASA15*) in Subfamily 1, four genes (*AcGASA4*, *AcGASA5*, *AcGASA8*, and *AcGASA13*) in Subfamily 2, and four genes (*AcGASA1*, *AcGASA9*, *AcGASA10*, and *AcGASA11*) in Subfamily 3. The distribution of pineapple *GASA* genes was predominant in Subfamily 1, which is like the distribution patterns observed in tobacco and grape *GASA* genes [4,6]. We observed that *AcGASA10* in pineapple clustered on the same small branch as *AcGASA9*, suggesting a closer evolutionary relationship between these two genes and their potentially shared functional roles.

### 2.5. Origins of the AcGASA Gene

**Figure 4:**
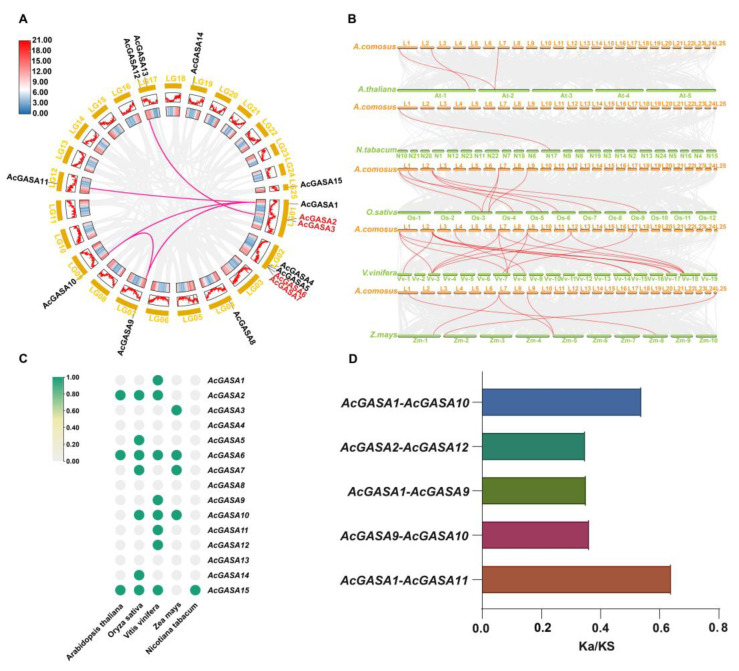
A collinear analysis of pineapple *AcGASA* genes and its homologs and the calculation of the Ka/Ks value of *AcGASA* genes. (**A**) Circos map of the pineapple GASA colinear homologous gene. Dark pink lines indicate colinear gene pairs, while tandem duplicated genes are highlighted in a red font. Each chromosome is presented as a 100 kb heat map depicting gene density. (**B**) The co-linearity of pineapple with *Arabidopsis thaliana*, tobacco, rice, grape, and maize; red lines indicate colinear gene pairs. (**C**) The heat map of nonredundant collinear *GASA* genes in pineapple and other species. (**D**) The bar graph shows the Ka/Ks ratio for the pineapple *AcGASA* gene.

Gene duplication is recognized as a significant driver of species evolution and a direct contributor to the expansion of gene families [44,45,46]. To investigate this phenomenon within the *AcGASA* gene, we conducted a comprehensive search for both tandem and segmental duplicates and presented the results using a Circos plot (Figure 4A). We observed that both tandem and segmental duplicates within the *AcGASA* gene family, suggesting their key role in the expansion of this gene family. We identified two pairs of tandem duplicates, comprising *AcGASA2* with *AcGASA3* and *AcGASA6* with *AcGASA7*, and five pairs of segmental duplicates, involving *AcGASA1* with *AcGASA10*, *AcGASA2* with *AcGASA12*, *AcGASA1* with *AcGASA9*, *AcGASA9* with *AcGASA10*, and *AcGASA1* with *AcGASA11* (Figure 4A).

To explore the ancestral relationship between pineapple *GASA* genes and their counterparts in various species, including pineapple, Arabidopsis thaliana (*A. thaliana*), tobacco (*N. tabacum*), rice (*O. sativa*), grape (*V. vinifera*), and maize (*Z. mays*), we conducted a comprehensive analysis of collinearity (Table S3). The pineapple *GASA* genes displayed three collinear gene pairs with Arabidopsis, while tobacco had 1 pair, rice had 9 pairs, grape had 18 pairs, and maize had 5 pairs (Figure 4B). There were no collinear relationships between *AcGASA4/8/13* in any of the species in our study (Figure 4C and Table S4). *AcGASA1*, *AcGASA9*, *AcGASA11*, and *AcGASA12* exhibited colinear relationships exclusively with the grape species (Figure 4C). On the other hand, *AcGASA3*, *AcGASA5*, and *AcGASA14* displayed colinear relationships with only one species (Figure 4C). *AcGASA6* and *AcGASA15* demonstrated the highest degree of colinearity among the identified pairs (Figure 4C). Subsequently, we calculated the Ka/Ks values (nonsynonymous/synonymous substitution ratio) for five duplicated *AcGASA* gene pairs (Figure 4D and Table S5). All *AcGASA* gene pairs had Ka/Ks values less than one, indicating that the *AcGASA* gene family evolved primarily under the influence of purifying selection.

### 2.6. Expression Profile of AcGASA Gene in Different Tissue Parts of Pineapple

**Figure 5:**
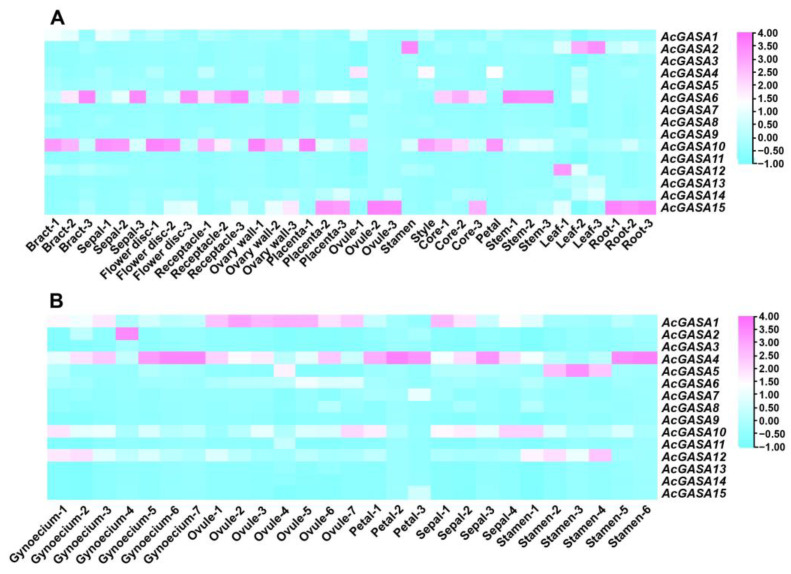
The expression of *AcGASA* genes within various tissue sites of pineapple. (**A**) The heat map of *AcGASA* gene expression in different tissue sites of pineapple across various developmental periods. (**B**) The heat map of *AcGASA* gene expression in pineapple floral organs (stamens, gynoecia, ovules, sepals, and petals) at different time points. There are five periods for stamens (S1–S5), seven periods for gynoecia (S1–S7), seven periods for ovules (S1–S7), four periods for sepals (S1–S4), and three periods for petals (S1–S3).

The expression of *AcGASA* genes was analyzed across various tissue types using pineapple transcriptome data from previous studies [47]. These tissue types included roots, stems, leaves, petals, stamens, pistils, ovules, ovaries, fruiting centers, bracts, sepals, flower discs, pedicels, and placentas (Table S6). Our findings revealed that most of the genes exhibited relatively low expression levels throughout the various pineapple tissues (Figure 5A). However, two genes, *AcGASA6* and *AcGASA10*, showed high expression patterns across multiple pineapple tissues (Figure 5A). *AcGASA10* displayed high expression during early pineapple development, whereas *AcGASA6* generally exhibited high expression in the later stages of pineapple development (Figure 5A).

We examined transcriptome data from prior studies and produced a gene expression heat map to gain insight into the expression patterns of *AcGASA* genes within floral organs (Figure 5B and Table S7) [48]. We observed that not all *AcGASA* genes exhibit high expression within these floral organs (Figure 5B). *AcGASA4* is highly expressed in almost the entire floral organ (Figure 5B). Interestingly, *AcGASA1* exhibits high expression levels during the entire development of ovules, while *AcGASA5* exhibited high expression levels during the initial and middle stages of stamen development (Figure 5B).

**Figure 6:**
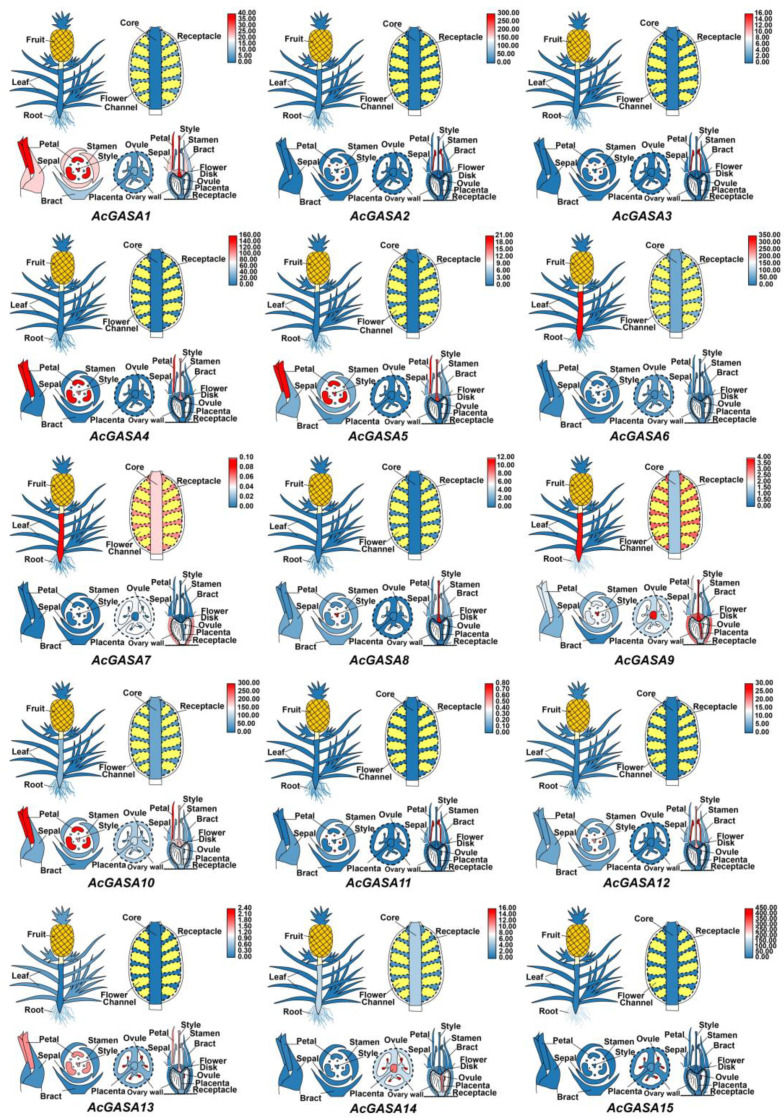
A cartoon heat map depicting the expression of *AcGASA* genes across various pineapple tissues. Each *AcGASA* gene expression level is represented by a pineapple cartoon heat map. Blue, white, and red colors indicate low, medium, and high levels of expression. These expression levels were computed by averaging the data for each site across different periods of transcriptome data.

To further visualize the expression patterns of *AcGASA* genes in various tissues, we used cartoon heat maps to display the expression levels of *GASA* genes (Figure 6 and Table S8). *AcGASA1*, *AcGASA4*, *AcGASA5*, *AcGASA10*, and *AcGASA13* exhibited high expression, primarily in petals and style (Figure 6). *AcGASA6*, *AcGASA7*, and *AcGASA9* demonstrated high expression in the roots (Figure 6). *AcGASA2*, *AcGASA3*, *AcGASA11*, and *AcGASA12* displayed high expression in the stamen (Figure 6). Among the analyzed tissues, *AcGASA7* was highly expressed in the core. In the placenta, we observed high expression levels of *AcGASA9* and *AcGAS14* (Figure 6). In the receptacle, *AcGASA7* and *AcGASA9* demonstrated a notably high expression (Figure 6). These distinct expression patterns across various tissue sites in pineapples underscore the potential role of *AcGASA* genes in the developmental processes of pineapples.

### 2.7. AcGASA Gene Protein Interactions Prediction and miRNA Target Prediction

**Figure 7:**
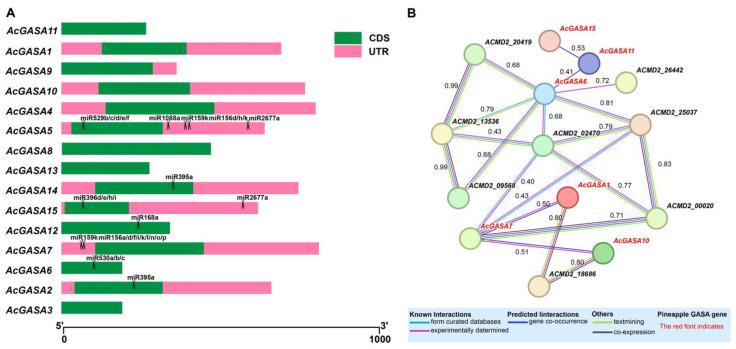
The prediction of miRNA target sites for *AcGASA* gene prediction and the protein interaction network prediction. (**A**) Predicted miRNA targets in the *AcGASA* gene. miRNAs target the *AcGASA* gene and are represented by scissors. (**B**) *AcGASA* protein interaction network using the pineapple protein as a reference.

We conducted a miRNA target prediction analysis for the *AcGASA* gene (Table S9), using established pineapple miRNAs as a reference [49]. Our analysis identified miRNA target sites in only seven genes, including *AcGASA2/5/6/7/12/14/15* (Figure 7A). We observed that the *AcGASA5* gene contains five miRNA target sites, with all four located within the 3’ UTR regions, except for one located in the CDS region (Figure 7A). *AcGASA15* has a miRNA target site within its 3’ UTR region, while *AcGASA7* presents two miRNA target sites in its 5’UTR region (Figure 7A). In *AcGASA2/6/12/14*, each gene possesses one miRNA target site located in the CDS region (Figure 7A).

An analysis of the predicted protein interaction networks indicates the presence of multiple interactions involving various *AcGASA* genes (Figure 7B), including serine/threonine protein kinase, phosphatase, and autophagy proteins. The *AcGASA* genes predicted to engage in interactions are *AcGASA1*, *AcGASA6*, *AcGASA7*, *AcGASA10*, *AcGASA11*, and *AcGASA15* (Figure 7B). These predicted interactions involve one or more partner proteins. Notably, GASA genes exhibit the following interacting relationships: *AcGASA15* with *AcGASA11, AcGASA11* with *AcGASA6*, *AcGASA7* with *AcGASA1*, *AcGASA7* with *AcGASA10* (Figure 7B). *AcGASA6* had the highest number of interactions among the predicted interactions (Figure 7B).

### 2.8. AcGASA Gene Promoter Analysis and Expression Patterns upon Different Hormone Treatments

**Figure 8:**
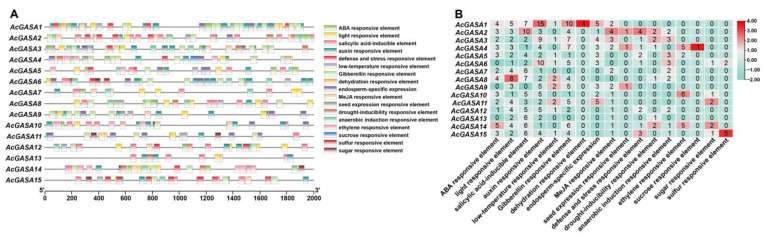
*AcGASA* gene promoter cis-elements analysis. (**A**) Promoter cis-element mapping analysis of the 2000 bp region upstream of the TSS (Transcription Start Site) of the *AcGASA* gene. (**B**) Heat map of the number of cis-elements of different promoters in the *AcGASA* gene.

To examine the functionality of the *AcGASA* gene, we conducted an analysis of cis-elements present in the 2000 bp sequence upstream of the Transcription Start Site (TSS) (Table S10). This allowed us to map the distribution of cis-elements across different *AcGASA* genes in pineapple (Figure 8A). We identified numerous hormone-responsive cis-elements, including the ABRE and CE3 elements, which are responsive to abscisic acid (ABA); the SRE, T/G-box, GCC-box, TGACG-motif, and CGTCA-motif elements responsive to methyl jasmonate (MeJA); the LS7, TCA, and W-box elements responsive to salicylic acid (SA); the ERE element responsive to ethylene; the NDE, D1, D2, and other auxin-responsive elements; and the GARE and P-box elements responsive to gibberellin (GA) (Table S10). In the promoters of *AcGASA7* and *AcGASA13*, GA response elements are absent (Figure 8B) in *AcGASAs*. Only a few *AcGASA* genes (*AcGASA4*, *AcGASA5*, *AcGASA10*, and *AcGASA14*) contain the ethylene response element, while the remaining *AcGASA* genes possess a significant number of other hormone-responsive elements. Furthermore, we identified various abiotic stress cis-elements, including light-responsive cis-elements (G-Box, LRE, TCT-motif, and MRE), defense and stress-responsive elements (TC-rich repeats, SRE, and CORE), low-temperature-responsive elements (LTR), dehydration-responsive elements (DRE), drought-induced responsive elements (MBS), and anaerobic-induced responsive elements (ARE and AGCAACGGTC motifs) (Figure 8A and Table S10). Additionally, we detected seed expression responsive elements (ACGT motif) and endosperm-specific expression elements (AACA motifs and GCN motifs) (Figure 8A and Table S10).

**Figure 9:**
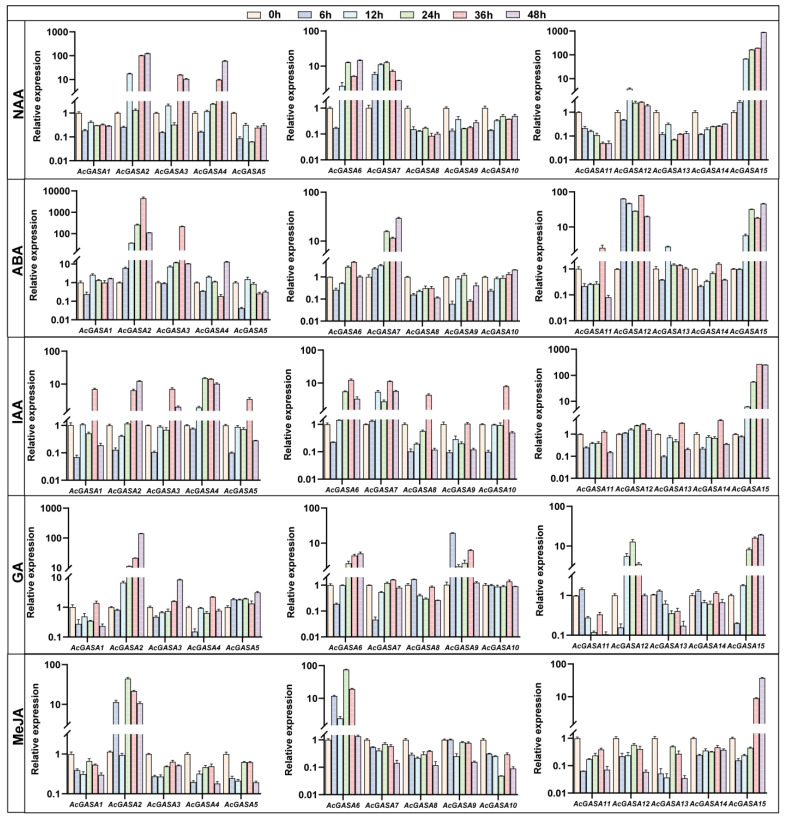
*AcGASA* gene expression profile under different hormone treatments. Expression levels of *AcGASA* genes in pineapple seedlings subjected to treatment with GA, ABA, NAA, IAA, and MeJA. The data presented are normalized to pineapple β-actin, and the error line represents the standard deviation calculated from three technical replicates.

We observed the significant presence of hormone-related cis-elements within the *AcGASA* genes. Subsequently, we detected the expression patterns of *AcGASA* genes in pineapple seedlings subjected to various hormonal treatments (GA, ABA, NAA, IAA, and MeJA) (Figure 9 and Table S11). All the *AcGASA* genes demonstrated hormone responsiveness. In NAA-treated pineapple seedlings, we observed a mixture of the repression and promotion of *AcGASA* genes. Specifically, eight genes (*AcGASA1/5/8/9/10/11/13/14*) exhibited downregulation, while seven genes (*AcGASA2/3/4/6/7/12/15*) displayed elevated expression levels (Figure 9). Following a 48 h treatment with MeJA, the majority of *AcGASA* gene expression in pineapple seedlings was repressed (Figure 9). Interestingly, despite the absence of MeJA-related cis-elements within the *AcGASA6* gene, the expression notably increased (Figure 8B and Figure 9). After 6 h of IAA treatment, most *AcGASA* genes showed repression, but most genes also displayed significant upregulation in expression after 36 h (Figure 9). Under GA treatment, a subset of *AcGASA* genes (*AcGASA1/4/6/7/12/15*) exhibited an initial phase of repression, but all genes displayed a significant upregulation in their expression, while another portion of genes (*AcGASA8/11/13/14*) exhibited upregulation followed by repression (Figure 9). We found that the *AcGASA2/3/7/12/15* expression was significantly upregulated after ABA treatment (Figure 9). These observations suggest that *AcGASA* genes play distinct roles under the influence of different hormones.

## 3. Discussion

Pineapple, a significant tropical fruit globally, is susceptible to various biotic and abiotic stresses that can profoundly impact its yield and quality. GASA proteins play a crucial role in the regulation of plant growth and development. While *GASA* genes have been extensively studied in several plant species, such as *Arabidopsis thaliana* [3], rice [5], grape [6], tobacco [4], and citrus [10], their presence in pineapple has not been previously documented. In this study, in pineapple, the *GASA* genes were identified. Their evolutionary relationships, along with promoter cis-elements and commonalities, were analyzed. Additionally, a comparison of sequence features and analyzed expression patterns was conducted under hormone treatments.

Through bioinformatic methods, we identified 15 *GASA* genes in pineapple. The pineapple *GASA* gene was categorized into three major subfamilies (Subfamily 1, Subfamily 2, and Subfamily 3), following the subfamily characterization observed in other species (*Arabidopsis thaliana*, tobacco) [4], which is a common practice in angiosperms [50]. To analyze the evolutionary relationships of the *GASA* gene in pineapple, we constructed a phylogenetic tree that included Arabidopsis, rice, maize, tobacco, and grape. Our analysis revealed that the *AcGASA* gene in pineapple primarily belongs to Subfamily 1, which is similar to the distribution in tobacco and grape [4,6]. This suggests that these genes may have experienced similar selective pressures during evolution, indicating shared biological functions and regulatory networks. To analyze the evolutionary relationships of *GASA* genes among different species, we constructed evolutionary trees of pineapple with Chlorophytes (2), Bryophytes (1), Pteridophytes (1), Gymnosperms (1), Basal angiosperms (1), Monocots (7), and Dicots (10). The number of *GASA* genes in pineapple is similar to that of other monocotyledonous plants. By contrast, dicotyledonous plants exhibit a higher number of *GASA* genes, and they are absent in *Chlorophyta* (*Chlamydomonas reinhardtii*, *Volvox carteri*), *Bryophyta* (*Physcomitrella patens*), and *Gymnosperm* (*Pinus taeda*). These findings substantiate the prevalence of *GASA* genes among land plants. Physicochemical analysis revealed that all *AcGASA* proteins demonstrate basic properties, with the majority being hydrophilic, except for *AcGASA2*, *AcGASA5*, and *AcGASA7*. These findings align with those reported in plum, tobacco, and Arabidopsis [4,51]. This suggests that AcGASA proteins tend to interact with other hydrophilic entities, including aqueous molecules and other proteins. This interplay might affect the functionality, folding conformation, and structural stability of the protein. Previous studies have detected *GASA* proteins in the cell membrane, cell wall, and nucleus of various plant species, corroborating our subcellular prediction results [5,15,52]. In addition, most *GASA* structural domains consist of 12 cysteines, which play a pivotal role in maintaining the spatial structure and function of GASA proteins [17,53]. *AcGASA13* does not possess the complete set of 12 conserved cysteines (Figure 1A), and the modifications or removal of essential peptide residues can render the GASA structural domains non-functional, leading to a loss of biological function. The limited variation in the number and arrangement of the 12 cysteines across different species throughout evolution strongly indicates their crucial role in plant activity.

We observed that the majority of *AcGASA* genes feature conserved motifs 1 and 2 (Figure 1D). The remaining conserved motifs seem relatively scarce. For instance, conserved motif 4 is exclusively found in *AcGASA7/12*, while conserved motif 5 is specifically present in *AcGASA8/13*. These alterations in conserved motifs imply that the function of the *AcGASA* gene exhibits evolutionary diversity. The *AcGASA* gene exhibits variations in the number of introns, ranging from 1 to 3 introns, and in exons, with 2–4 per gene. This suggests that changes in exons and introns could be attributed to chromosome modifications [54,55]. We hypothesized that variations in the number and length of introns and exons could contribute to divergent biological functions of the *AcGASA* gene. However, the intron numbers and lengths in the pineapple *GASA* genes were not conserved, even within the same subfamily (Figure 1F). Additionally, the *AcGASA* genes were found to be randomly distributed throughout the genome (Figure 1B). These observations suggest the absence of recent duplication events in the *GASA* gene family and indicate that the expansion of this gene family is a result of earlier duplication events.

Segmental, tandem and genome-wide duplications are significant driving forces shaping the evolution of gene families [45,56]. Segmental and tandem duplications exert a more pronounced influence on the functional evolution of gene families [57]. In the case of the *AcGASA* gene family in pineapple, both segmental and tandem duplications are prominent, with the identification of five sets of segmental duplications and two sets of tandem duplications. The prevalence of segmental duplications aligns with previous studies that highlight their higher occurrence in gene families compared to tandem duplications [58]. Furthermore, when examining the collinearity of *GASA* genes between pineapple, *Arabidopsis thaliana*, tobacco, maize, rice, and grape, it becomes evident that there is a greater number of covariant gene pairs between pineapple and grape. This observation suggests a closer evolutionary relationship between pineapple and grape in comparison to the other plants studied. Interestingly, pineapple and tobacco exhibit only one pair of collinear genes, implying a higher occurrence of genetic rearrangement events or a more extended period of divergence during their evolution. By identifying nonredundant *GASA* genes with the collinear relationship between pineapple and other five species, we found that *AcGASA1/9/11/12* has direct homologs exclusively in grapes, while *AcGASA3* has direct homologs in maize and *AcGASA5/14* has direct homologs in rice. This indicates the variability in the preservation of *GASA* gene family members among species with distinct evolutionary backgrounds. The calculation of Ka/Ks ratios for the *AcGASA* genes in pineapple reveals that all of them fall below one, implying that the pineapple *GASA* genes were subjected to robust purifying selection throughout their evolutionary history.

The analysis of cis-elements in gene promoter regions plays a pivotal role in understanding gene function [59]. Within the *AcGASA* promoter of pineapples, we identified numerous elements that respond to hormones, promote growth and development, and respond to stress. Among these elements, GA, MeJA, SA, and auxin response elements are highly abundant in the promoter region of the *AcGASA* gene. Notably, we observed that the dehydration response element, sucrose response element, and sucrose response element were identified in only a limited number of genes, indicating that certain *AcGASA* genes can exhibit specific responses and regulatory mechanisms toward these three stresses. Conversely, other genes may rely on distinct regulatory pathways or possess alternative response elements. This underscores the diversity and variability inherent within gene families, highlighting the intricate regulatory networks involved in plant adaptation to stress conditions. Upon treating pineapple seedlings with various hormones, we observed varying degrees of gene regulation across all genes. The *AcGASA2* and *AcGASA15* genes exhibited significant expression levels 48h after hormone treatment (NAA, IAA, ABA, GA, and MeJA), and these findings can serve as a reference point for future *AcGASA* gene studies. The response of *GASA* genes to ABA differs across different species. In Arabidopsis, ABA leads to an increase in the expression of *AtGASA2/3/5/14*, while it causes a decrease in the expression of *AtGASA7/9* [30]. In *Nicotiana tabacum*, *NtGASA1/2/3/4/8/9/13/14* is induced by ABA, as is *PmGASA1/2/6/11/12/14* in *Prunus mume* [3,51]. In our study with pineapple seedlings, we found that ABA strongly upregulates *AcGASA2/3/4/6/7/10/12/13/15* over a period. Interestingly, *AcGASA8* contained 4ABA-responsive elements but saw a significant inhibition of its expression. In Arabidopsis, *AtGASA1* is induced by GA but inhibited by ABA [37]. We observed a similar pattern in pineapple seedlings. For example, after 6 h of hormone treatment, GA induced the expression of *AcGASA11*, which was subsequently inhibited by ABA. In contrast to the response in other species, GA treatment induced the *GASA* gene in potato and tobacco seedlings. However, in pineapple seedlings, only *AcGASA2/4/6/9/12/15* was highly induced over time [4,7]. While NAA significantly reduced *OsGASA* gene expression in rice seedlings, only specific genes (*AcGASA5/8/9/10/13/14*) were downregulated in pineapple [18]. Additionally, we found that most *AcGASA* genes were inducible by IAA, with only *AcGASA2/6/15* exhibiting a significant induction in response to MeJA. This distinct expression pattern under hormone treatment in the *AcGASA* gene in pineapple sets it apart from other species, indicating the crucial function of *AcGASA* genes in regulating the growth and development of pineapples.

In addition to its hormonal responses, *AcGASA* plays a crucial role in the growth and development of various plant tissues. To clarify the functions and roles of the *AcGASA* gene across various parts of pineapple, we conducted an examination of its expression patterns in various tissue organs. Some genes, like *AcGASA6* and *AcGASA10*, displayed universal expression patterns similar to *NtGASA11* and *NtGASA17* in tobacco [4]. Some studies have reported that GASA genes have flower-inducing effects in various plant species, such as petunia, rice, cotton, and Gerbera hybrida [17,60,61,62]. Most *AcGASA* genes exhibit significantly higher expression levels in their floral organ tissues, similar to the patterns in *NtGASA* genes in tobacco. We identified genes with a notably high expression in specific tissue types, such as *AcGASA6*, *AcGASA7*, *AcGASA15* in the roots, *AcGASA2* and *AcGASA12* in the leaves, and *AcGASA6* and *AcGASA7* in fruit pulp. The diverse expression of the *AcGASA* gene across various tissues indicates distinct functions in the growth and development of pineapple plants.

## 4. Materials and Methods

### 4.1. Protein Sequence Source

The genome sequences used in this study were obtained from the Pineapple Genome Project [63]. The sequences of GASA proteins from Arabidopsis thaliana, rice, grape, and tobacco were acquired in previous studies [4]. The protein sequences of *Physcomitrella patens* and *Pinus taeda* were retrieved from the PlantGenIE database (https://plantgenie.org/, accessed on 5 October 2023). Protein sequences from additional species, including corn and soybean, were obtained through Phytozome (http://www.phytozome.net/, accessed on 5 October 2023).

### 4.2. Identification and Classification of GASA

For characterizing the GASA gene in pineapple, we employed two distinct methods. We retrieved the Hidden Markov Model for the GASA structural domain (PF02704) from the Pfam database (http://pfam.xfam.org/, accessed on 7 October 2023) and subsequently queried the pineapple protein file using TBtools and applying an E-value of 1e-10 [64]. Next, we conducted a focused BlastP search, utilizing Arabidopsis GASA protein sequences against pineapple protein sequences, leading to the identification of 15 potential GASA candidates. To confirm the presence of GASA-conserved structural domains, we utilized both Batch CD-Search and SMART programs. We applied this methodology to detect the *GASA* gene in 19 additional species, including corn and soybean. Further analyses involved assessing the physicochemical properties of AcGASA genes/proteins using the ExPASy ProtParam database (https://web.expasy.org/protparam/, accessed on 7 October 2023).

Additionally, we predicted the subcellular localization of the *AcGASA* gene through Plant-mPLoc (http://www.csbio.sjtu.edu.cn/bioinf/plant-multi/, accessed on 7 October 2023). Finally, we analyzed the evolutionary relationships of 23 species by utilizing the online website TimeTree (http://timetree.org/, accessed on 7 October 2023) and calculated the number of GASA genes in each species.

### 4.3. Gene Structure and Conserved Motif Analysis of the AcGASA Gene

We employed MEME to analyze the conserved motifs within the AcGASA protein [65]. To further understand the genomic context, we analyzed the pineapple genome annotation using TBtools [63,64]. Subsequently, TBtools were used to represent the exons, introns, and conserved motifs in *AcGASA*.

### 4.4. Collinear Analysis and Chromosomal Location of the AcGASA Gene

To determine the position of the *AcGASA* gene on the pineapple chromosome and highlight tandem and segmental repeats, we conducted a collinear analysis using the "One Step MCScanX Wrapper" plug-in on TBtools [64]. This analysis revealed gene pairs displaying both segmental and tandem duplications, which were depicted in a Circos plot. Moreover, we conducted a collinear analysis of pineapple with other species, such as tobacco, maize, rice, grape, and Arabidopsis thaliana, using TBtools for visualization.

### 4.5. Analysis of the Cis-Elements of the AcGASA Gene Promoter

To conduct a comprehensive investigation into the functional attributes of the *AcGASA* gene, we obtained a 2000 bp promoter sequence upstream of the gene’s TSS (Transcription Start Site) from Phytozome (http://www.phytozome.net/, accessed on 13 November 2023). These promoter sequences were then submitted to the online platform PlantPAN 4.0 (http://plantpan.itps.ncku.edu.tw/plantpan4/promoter_analysis.php, accessed on 13 November 2023) for prediction and analysis [59]. Finally, we employed Tbtools to select and visualize the pertinent cis-elements.

### 4.6. Protein Interaction Network and miRNA Target Prediction of the AcGASA Gene

We submitted all *AcGASA* gene protein sequences to the online STRING website (http://string-db.org, accessed on 10 October 2023). Within this network, pineapple was selected as the reference, and only the highest-scoring proteins with interactions were retained. Those without protein interactions were subsequently removed.

We used the published pineapple miRNA as a reference [39], submitted the AcGASA sequence to the online website psRNATarget (https://www.zhaolab.org/psRNATarget/, accessed on 10 October 2023) for predicting miRNA targets, and finally visualized it using TBtools [66].

### 4.7. Expression Map of the AcGASA Gene in Different Pineapple Tissues

From published studies, we obtained transcriptomic data from various tissues of pineapple [47,48]. These transcriptome datasets were downloaded from NCBI and associated with project numbers PRJEB38680 and PRJNA483249. The expression levels of the *AcGASA* gene were analyzed using FPKM values, and we generated an expression heatmap and cartoon heatmap using TBtools [64].

### 4.8. Hormone Treatment of Pineapple Seedlings and AcGASA Expression Analysis

Pineapple seedlings underwent treatment with 0.1 mM indole-3-acetic acid (IAA), abscisic acid (ABA), NAA, GA, and MeJA. Samples were collected at 0, 6, 12, 24, 36, and 48 h. Each sample was treated with liquid nitrogen and promptly stored at −80 °C after collection.

The total RNA extraction was carried out using the FastPure Universal Plant Total RNA isolation Kit (Vazyme, Nanjing, China). The RNA quality was assessed through agarose gel electrophoresis, and RNA concentration was determined using a Nanodrop ND-1000spectrophotometer. The first-strand cDNA synthesis was obtained from total RNA using the All-in-One First-Strand Synthesis MasterMix (Guangzhou XinKaiLai Biotechnology Co, Guangzhou, China). Primer design was accomplished using the online URL Primer3Plus (https://www.bioinformatics.nl/cgi-bin/primer3plus/primer3plus.cgi, accessed on 13 October 2023). The qRT-PCR analysis of the AcGASA gene was performed on a Roche LightCycler 480 instrument using SYBR Green Master Mix (YEASEN). The total qRT-PCR reaction system consisted of 10 μL, comprising 5 μL of the Hieff qPCR SYBR Green Master Mix, 0.4 μL of the Forward and Reverse Primer (10 μM), 1 μL of the cDNA template, and 3.6 μL of ddH2O. The reaction parameters were set at 95 °C for 5 min, followed by 40 cycles of 95 °C for 10 s and 60 °C for 30 s. Each reaction was performed with three technical replicates. Relative expression was calculated using the 2^−ΔΔCt^ method, with pineapple β-actin serving as the internal reference gene. The primers used for qRT-PCR experiments are shown in Table S12.

## 5. Conclusions

In this study, we identified and analyzed 15 GASA genes in pineapple, including their chromosomal locations, gene structures, conserved motifs, and the physicochemical properties of the corresponding proteins. The phylogenetic tree analysis revealed three main branches. The subcellular localization prediction of the *AcGASA* gene indicated its presence in multiple cellular compartments, including the cell membrane, Golgi apparatus, nucleus, and cell wall. Collinearity analysis demonstrated that segmental and tandem duplications played a crucial role in expanding the pineapple GASA gene family, and purifying selection was the primary evolutionary force acting on the *AcGASA* gene. Promoter analysis identified a diverse array of cis-elements, including hormone-responsive and stress-responsive elements. In particular, the *AcGASA* gene exhibited a significant abundance of hormone response elements, such as GA response elements, ABA response elements, SA response elements, and MeJA response elements. Furthermore, we examined the spatiotemporal expression patterns of the *AcGASA* gene in various pineapple organs and during hormonal responses. Our results show the distinct expression patterns of the *AcGASA* gene during the development of different tissues in pineapple, particularly during floral organ tube development. These findings suggest that *AcGASA* genes are differentially regulated by phytohormones such as GA, NAA, IAA, MeJA, and ABA. This study provides valuable insights into the role and regulatory mechanisms of *AcGASA* genes in pineapple growth and development and can serve as a reference for future investigations on the functional characteristics of GASA genes in other perennial herbaceous plant species.

## Figures and Tables

**Table 1:** The physicochemical properties of the *AcGASA* family genes. For each gene, the table provides the gene ID, protein length (PL), molecular weight of amino acid (MW), isoelectric points (pI), hydrophilicity score (GRAVY), aliphatic index (AI), and the predicted subcellular location.

Gene Name	Gene ID	PL (aa)	MW (kDa)	pI	GRAVY	AI	Subcellular Localization
*AcGASA1*	Aco012370	90	10.08	9.07	−0.149	64.00	Cell membrane, Golgi, nucleus
*AcGASA2*	Aco011225	94	10.17	8.48	0.007	72.66	Golgi
*AcGASA3*	Aco011224	63	6.96	9.16	−0.805	17.14	Golgi, nucleus
*AcGASA4*	Aco001116	111	12.35	8.84	−0.307	56.31	Golgi
*AcGASA5*	Aco001067	95	10.3	8.98	0.014	58.53	Cell membrane, Golgi, nucleus
*AcGASA6*	Aco000980	63	6.67	8.96	−0.398	29.52	Golgi, nucleus
*AcGASA7*	Aco000979	116	12.37	8.59	0.011	69.14	Cell wall
*AcGASA8*	Aco002373	155	17.39	9.25	−0.691	45.42	Nucleus
*AcGASA9*	Aco004893	95	10.72	9.20	−0.229	74.00	Cell membrane, Golgi, nucleus
*AcGASA10*	Aco008536	97	10.69	8.98	−0.070	71.44	Cell membrane, cell wall, Golgi
*AcGASA11*	Aco000098	88	9.85	8.88	−0.365	54.43	Golgi
*AcGASA12*	Aco003244	114	12.51	9.23	−0.104	65.96	Golgi
*AcGASA13*	Aco015815	93	10.54	9.17	−0.932	38.92	Golgi, nucleus
*AcGASA14*	Aco015632	106	11.52	8.65	−0.010	73.77	nucleus
*AcGASA15*	Aco010336	67	7.12	8.79	−0.482	35.07	Golgi, nucleus

## Data Availability

The datasets used in this study are publicly available. All analyzed data can be found in the article or in the Supplementary Material.

## Supplementary Materials

The following supporting information can be downloaded at: https://www.mdpi.com/article/10.3390/ijms242317063/s1.
