# Supplementary material for: Genome-Wide Identification and Characterization of Gibberellic Acid-Stimulated Arabidopsis Gene Family in Pineapple (Ananas comosus)
Source: Int J Mol Sci. 2023 Dec 2;24(23):17063. doi: 10.3390/ijms242317063 (PMC10706908; doi:10.3390/ijms242317063)
Supplement: Supplementary file 1 [file ijms-24-17063-s001.zip › Table S1.pdf]

Table S1: Physicochemical properties of *AcGASA* family genes. Each gene is presented with its respective Gene ID, Chromosomal locations, length of coding region (CDS), Protein length (PL), Molecular weights of amino acid (MW), Isoelectric points (pI), Hydrophilicity score (GRAVY), Aliphatic index (AI), and predicted subcellular location.

Gene name	Gene ID	Chr location	CDS (bp)	PL (aa)	MW (kDa)	pI	GRAVY	AI	Subcellular localization
AcGASA1	Aco012370	LG01:2575624-2576905	273	90	10.08	9.07	-0.149	64.00	Cell membrane, Golgi, Nucleus
AcGASA2	Aco011225	LG01:13308868-13310528	285	94	10.17	8.48	0.007	72.66	Golgi
AcGASA3	Aco011224	LG01:13310793-13311280	192	63	6.96	9.16	-0.805	17.14	Golgi, Nucleus
AcGASA4	Aco001116	LG02:14120832-14122260	336	111	12.35	8.84	-0.307	56.31	Golgi
AcGASA5	Aco001067	LG02:14490031-14490928	288	95	10.3	8.98	0.014	58.53	Cell membrane, Golgi, Nucleus
AcGASA6	Aco000980	LG02:15218075-15218487	192	63	6.67	8.96	-0.398	29.52	Golgi, Nucleus
AcGASA7	Aco000979	LG02:15218868-15219954	351	116	12.37	8.59	0.011	69.14	Cell wall
AcGASA8	Aco002373	LG04:2068331-2070499	468	155	17.39	9.25	-0.691	45.42	Nucleus
AcGASA9	Aco004893	LG07:593092-593647	288	95	10.72	9.20	-0.229	74.00	Cell membrane, Golgi, Nucleus
AcGASA10	Aco008536	LG09:569695-571352	294	97	10.69	8.98	-0.070	71.44	Cell membrane, Cell wall, Golgi
AcGASA11	Aco000098	LG12:4718773-4719394	267	88	9.85	8.88	-0.365	54.43	Golgi
AcGASA12	Aco003244	LG17:1390314-1391450	345	114	12.51	9.23	-0.104	65.96	Golgi
AcGASA13	Aco015815	LG17:5770304-5771114	282	93	10.54	9.17	-0.932	38.92	Golgi, Nucleus
AcGASA14	Aco015632	LG19:1022203-1025827	321	106	11.52	8.65	-0.010	73.77	Nucleus
AcGASA15	Aco010336	LG25:1950442-1951286	204	67	7.12	8.79	-0.482	35.07	Golgi, Nucleus
