# Supplementary material for: Genome-Wide Identification and Characterization of Gibberellic Acid-Stimulated Arabidopsis Gene Family in Pineapple (Ananas comosus)
Source: Int J Mol Sci. 2023 Dec 2;24(23):17063. doi: 10.3390/ijms242317063 (PMC10706908; doi:10.3390/ijms242317063)
Supplement: Supplementary file 1 [file ijms-24-17063-s001.zip › Table S12.pdf]

Table S12: The primers of AcGASA genes and β-actin gene used in the qRT-PCR assays.

Gene	Primer	Sequence
AcGASA1	F	CGTCGAGTACTGCAACCTGT
	R	GGTTTACCCTTGCTGTTGCG
AcGASA2	F	GTGTTGGCTCAGGGAACAGA
	R	GGGGCATTGATAGCTTCGGA
AcGASA3	F	TGAATGAAGGGTCTGCAATAGA
	R	AAGGGCAGACCTCGTAGTTG
AcGASA4	F	TGATTGTCCTGCAAAGTGCG
	R	TCTGTGTTCCCAGAGGTTCC
AcGASA5	F	GCTCCTGCACCTCACAAAAT
	R	TGGGTCTGCCAATCAGTGTA
AcGASA6	F	AGCTCCTCTACACCGCGTTA
	R	ACCTGTCCTTGTTGCCGTAG
AcGASA7	F	CAAAGTGCGACTACCGATGC
	R	AGCATAGCAAGGGCAGACAT
AcGASA8	F	CCTGTAAAACGTGCTGTGCG
	R	GCAGGGGCACTGATCGTAAT
AcGASA9	F	ATAAACTGTGCGGGGCTGT
	R	TAAGGGCACTTGGTCCTGTT
AcGASA10	F	CTGGCTCAGCATTTTGCGAC
	R	CGGTGTACTTGTCGCGGTAG
AcGASA11	F	AAGCAGTGCACCAGACACTT
	R	TTGATAGGCTGCGGAGTTGG
AcGASA12	F	GAAGGCTCAGTTCCTCCAGA
	R	CCTCCTTCGTCTTCCAGTTG
AcGASA13	F	TCCCGGAGACCAATAATCAA
	R	GCACTTGGGTTTGAGTCCAT
AcGASA14	F	GGAGGGAATCTCAACCCTTC
	R	ACCCCTCTTGGTCTTCCAGT
AcGASA15	F	CACCTTGGCCCTCATACTGC
	R	ATGCATCGCCCTGTCTTCC
β-actin	F	CTGGCCTACGTGGCACTTGACT
	R	CACTTCTGGGCAGCGGAACCTTT
