# Supplementary material for: Genome-Wide Identification and Characterization of Gibberellic Acid-Stimulated Arabidopsis Gene Family in Pineapple (Ananas comosus)
Source: Int J Mol Sci. 2023 Dec 2;24(23):17063. doi: 10.3390/ijms242317063 (PMC10706908; doi:10.3390/ijms242317063)
Supplement: Supplementary file 1 [file ijms-24-17063-s001.zip › Table S2.pdf]

Table S2: The GASA protein sequences of Pineapple, *Arabidopsis*, Maize, Tobacco, Grape, and Rice.

Pineapple GASA proteins

>AcGASA1
MKLLPYTLALILLLLLTSSYLQATMALSDFCNSKCQGRCAKAGKTGRCMRYCGMCCEQCK
CVPSGTYGNKDECPCYRDMVSWKTKRPKCP*
>AcGASA2
MANKARFLSPVLLLLLLVEMNIFDGPLVQGVEGIDCAAKCDYRCSKAGWHKACIRACNTC
CERCHCVPPGTSGNKDVCPCYANMTTHGGRPKCP*
>AcGASA3
MRDDCPAKCAYRCSKSGRPKMCLRACNTCCQRCHCVPPGTSGNTEKCPCWAHMKTHGG
RPKCP*
>AcGASA4
MAMAYKLFILVAFALLAISMAEDKVLAQGTDHVEGEYHLEGSNGQGSLRSYQCPGQCSRR
CAQTQYKKPCLFFCQKCCMKCLCVPSGYYGNKGECPCYNNWKTKRGGPKCP*
>AcGASA5
MVVAAGFSRFSCSLLLLLLLALSFSESMGGNLNPSDCNPRCSYRCSGTSHKKPCMSFCMQC
CAQCLCVPPGTYGNRQVCACYNNWKTKRGGPKCP*
>AcGASA6
MINCAGLCAGRCSQHSRPNLCARACGTCCSRCKCVPPGTSGNREMCGTCYTNMTTHGN
RTKCP*
>AcGASA7
MALKLALFLLACFLLASTRVSSDSEEAFTEAAMAPYVPATPPALAPAPHKIIDTKECVGACK
ERCSLHSRKNVCSRACLTCCSVCKCVPAGTAGNKETCGKCYTDWQTHGNRTKCP*
>AcGASA8
MATTKQTKEAVHQTLHMDLERLLLQYLQQRANATRRAQKTTQQMFPEAQLRDNGMLIA
KHCPSPTSQPPAISPSPTPQPIKFPMYGVTPGSLHPQECPDRCAGRCSATAYKKPCMFFCQK
CCEKCLCVPPGTYGNKQFCPCYNDWKTKRGGPKCP*
>AcGASA9
MKLLYTALPLLLLLLLLLLASDHFQATMAGSAFCNGKCKVRCSKASEQERCLKYCGLCCEE
CRCVPPGTYGNKDRCPCYRDKFTGHGRRRRPKCP*
>AcGASA10
MKPLYITTTTTLAFLLLLLLLASSYLQTSMAGSAFCDGKCKVRCSKASVQDRCLRYCGLCCA
ECNCVPSGTYGNKDECPCYRDKYTGTGLRRRSKCP*
>AcGASA11
MKLFSFSLALMMLLLASAYLQTFINASEWCEGKCSERCSKAGARRRCVEYCNLCCKECNC
VPSGTAGNKDECPCYRDKRNSKGKPKCP*
>AcGASA12
MDSGRNKKLVSWFIVLFLFMQVVAEASLHNFIGLEDDKEGNATPADGFSRRPIINCNFACA
RRCMKASRKNVCARACGTCCIKCQCVPAGTSGNKTMCPCYANLRTHGLKPKCP*
>AcGASA13
MNRDRSPKISKPNRNSLAEIIGGANASHYLPAEKAQFLQKYRCSATSHKKPCLFFCNECCQ
KCHCVPSGTYGHKEECPCYNNWKTKEGKPKCP*
>AcGASA14

MATTQLILLTLLLAFSFTLHHLAVEAHEEVEHQLASKGARTLLATMDCNSACSGRCQLSSR
LNYCMRACKTCCARCSCVPPGTSGNYDQCPCYASLTTRSSKKKCP*
>AcGASA15
MNEGSAIDCGGACAERCKESSRPNLCKRACGTCCRRCSCVPPGTYGNYEVCPCYAAITTR
GGRKKCP*

Arabidopsis thaliana GASA proteins
>AtSA1
MAISKALIASLLISLLVLQLVQADVENSQKKNGYAKKIDCGSACVARCRLSRRPRLCHRACGTCCYRCNCVP
PGTYGNYDKCQCYASLTTHGGRRKCP
>AtSA2
MAVFRSTLVLLLIIVCLTTYELHVHAADGAKVGEGVVKIDCGGRCKDRCSKSSRTKLCLRACNSCCSRCNCV
PPGTSGNTHLCPCYASITTHGGRLKCP
>AtSA3
MAIFRSTLVLLLILFCLTTFELHVHAAEDSQVGEGVVKIDCGGRCKGRCSKSSRPNLCLRACNSCCYRCNCV
PPGTAGNHHLCPCYASITTRGGRLKCP
>AtSA4
MAKSYGAIFLLTLIVLFMLQTMVMASSGSNVKWSQKRYGPGSLKRTQCPSECDRRCKKTQYHKACITFCN
KCCRKCLCVPPGYYGNKQVCSCYNNWKTQEGGPKCP
>AtSA5
MANCIRRNALFFLTLLFLLSVSNLVQAARGGGKLKPQQCNSKCSFRCSATSHKKPCMFFCLKCCKKCLCVP
PGTFGNKQTCPCYNNWKTKEGRPKCP
>AtSA6
MAKLITSFLLLTILFTFVCLTMSKEAEYHPESYGPGSLKSYQCGGQCTRRCSNTKYHKPCMFFCQKCCAKCL
CVPPGTYGNKQVCPCYNNWKTQQGGPKCP
>AtSA7
MKIIVSILVLASLLLISSSLASATISDAFGSGAVAPAPQSKDGPALEKWCGQKCEGRCKEAGMKDRCLKYCGI
CCKDCQCVPSGTYGNKHECACYRDKLSSKGTPKCP
>AtSA8
MKLVVVQFFIISLLLTSSFSVLSSADSSCGGKCNVRCSKAGQHEECLKYCNICCQKCNCVPSGTFGHKDECP
CYRDMKNSKGGSKCP
>AtSA9
MKKMNVVAFVTLIISFLLLSQVLAELSSSSNNETSSVSQTNDENQTAAFKRTYHHRPRINCGHACARRCSKT
SRKKVCHRACGSCCAKCQCVPPGTSGNTASCPCYASIRTHGNKLKCP
>AtSA10
MKFPAVKVLIISLLITSSLFILSTADSSPCGGKCNVRCSKAGRQDRCLKYCNICCEKCNYCVPSGTYGNKDEC
PCYRDMKNSKGTSKCP
>AtSA11
MAVFRVLLASLLISLLVLDFVHADMVTSNDAPKIDCNSRCQERCSLSSRPNLCHRACGTCCARCNCVAPG
TSGNYDKCPCYGSLTTHGGRRKCP
>AtSA12
MMKLIVVFVISSLLFATQFSNGDELESQAQAPAIHKNGGEGSLKPEECPKACEYRCSATSHRKPCLFFCNKC
CNKCLCVPSGTYGHKEECPCYNNWTTKEGGPKCP
>AtSA13

MALSLLSVFIFFHVFTNVVFAASNEESNALVSLPTPTLPSPSPATKPPSPALKPPTPSYKPPTLPTTPIKPPTTKP
PVKPPTIPVTPVKPPVSTPPIKLPPVQPPTYKPPTPTVKPPSVQPPTYKPPTPTVKPPTTSPVKPPTTPPVQSPP
VQPPTYKPPTSPVKPPTTTPPVKPPTTTPPVQPPTYNPPTTPVKPPTAPPVKPPTPPPVRTRIDCVPLCGTRC
GQHSRKNVCMRACVTCCYRCKCVPPGTYGNKEKCGSCYANMKTRGGKSKCP
>AtSA14
MKMPVVVQFFIISLLLTSSFYVLSSADSSACGGKCSVRCSKADRTHEECLEDCDICCQKCNCVPSGTYGNKD
ECPCYRDMKNSKGGSKCP
>AtSA15
MATKLSIIVFSIVVLHLLLSAHMHFLINVCAECETKSAIPPLLECGPRCGDRCSNTQYKKPCLFFCNKCCNKCL
CVPPGTYGNKQVCPCYNNWKTKSGGPKCP

Maize GASA proteins
>ZmGASA1
MTMTTMKKQQQLLLLLSLMFLVAVTAAAVAADPHPQQVQVQQQQQAQMRINRATRSLL
PQ
PPPKLDCPSTCSVRCGNNWKNQMCNKMCNVCCNKCSCVPPGTGQDTRHLCPCYDTML
NPH
TGKLKCP*
>ZmGASA2
MAQASSSFSIVLLFLALVLVVEVSAGTANEELYRPAGAEGSVPIEQCPEKCDYRCSATSY
KKPCLFFCNYCCNKCLCVPSGTYGNKEECPCYDNMKTKQGGPKCP*
>ZmGASA3
MCMCSAATLPCCHVGRHGHQLHCTAPPHSYIVYCCCAHSLYICITACFWNQERRSPLHSR
RHPPLPLLFLEMTMTTMKKQQQLLLLLSLMFLVAVTAAAVAADPHPQQVQVQQQQQAQ
M
RINRATRSLLPQPPPKLDCPSTCSVRCGNNWKNQMCNKMCNVCCNKCSCVPPGTGQDTR
H
LCPCYDTMLNPHTGKLKCP*
>ZmGASA4
MAKPPLQTAAIILLVLLAAASCLHTVDAATLGFCWGKCSVRCAGATARQARAACMSSCGL
CCEACNCVPHDIHDCPCYRNMLTAGPKKRPKCP*
>ZmGASA5
MAAASGRAPSACALLLLFLLLVVGAAAAAVIVVDANRGEQEQDWDWEQLSAASPSPWSP
A
PAPAPSPVSFIDCGSACGARCALSSRWNLCRRACGSCCARCNCVPPGTAGNHDVCPCYAA
ITTRGGRPKCP*
>ZmGASA6
MAMAKPPLQTVTIILLVLLTAASWLHTVDASALGFCWGKCSVRCVQATAGQARAACMSS
Y
GLCCKACNCVPHDIHDCPCYRNMPTVDPKKRPKCP*
>ZmGASA7
MAKASSRLLFSLSLVVLLLLVETTTSPHGQADAIDCGASCSYRCSKSGRPKMCLRACGTC
CQRCGCVPPGTSGNEDVCPCYANMKTHDGQHKCP*
>ZmGASA8

MAVAKPPLQTAAVLLLLLLLVVAAASWLQTVDAASGFCSSKCSVRCGRAASARARGACMRS
CGLCCEECNCVPTRPPRDVNECPCYRDMLTAGPRKRPKCP*
>ZmGASA9
MAKPPLQTAAIILLVLLAAASCLHTVDAAALGFCWGKCSVRCAGATARQARAACMSSCG
L
CCEACNCVPRDIHDCPCYRNMLTAGPKKRPKCP*
>ZmGASA10
MRINRATRSLLPQPPPKLGTTENCRQQDTRSHFIDACFSRNTDCPSTCSVRCGNNWKNQM
CNKMCNVCCNKCSCVPPGTGQDTRHLCPCYDTMLNPHTGKLKCP*
>ZmGASA11
MTMTTMKKQQQLLLLLSLMFLVAVTAAAVAADPHPQQVQVQVQQQQQAQMRINRATRS
LL
PQPPPKLDCPSTCSVRCGNNWKNQMCNKMCNVCCNKCSCVPPGTGQDTRHLCPCYDT
MLNPHTGKLKCP*
>ZmGASA12
MTMTTMKKQQQLLLLLSLMFLVAVTAAAVAADPHPQQVVQVQQQQQAQMRINRATRSL
LP
QPPPKLDCPSTCSVRCGNNWKNQMCNKMCNVCCNKCSCVPPGTGQDTRHLCPCYDTM
LNP
HTGKLKCP*
>ZmGASA13
MTMTTMKKQQQLLLLLSLMFLVAVTAAAVAADPHPQQVQVQVQQQQQAQMRINRATRS
LL
PQPPPKLGTTENCRQQDTRSHFIDACFSRNTDCPSTCSVRCGNNWKNQMCNKMCNVCC
NK
CSCVPPGTGQDTRHLCPCYDTMLNPHTGKLKCP*
>ZmGASA14
MASRNKAAALLLCFLFLAAVAASAAEMIAGSGIGDGEGEELDKGGGGGGGHHKHEGYK
NK
DGKGNLKPSQCGGECRRRCSKTHHKKPCLFFCNKCCAKCLCVPPGTYGNKETCPCYNNW
K
TKKGGPKCP*
>ZmGASA15
MKPAATARVAGLLFFLVLLLALPSLRVSMAGSGFCDGKCAVRCSKASRHDDCLKYCGICC
ATCNCVPSGTAGNKDECPCYRDMTTGHGNRTRPKCP*
>ZmGASA16
MAPSKLAVVVALVASLLLLTTSNTKLGLFVLGQAAPGAYPPRAPPPHQIVDLAKDCGGAC
DVRCGAHSRKNICTRACLKCCGVCRCVPAGTAGNQQTCGKCYTDWTTHGNKTKCP*
>ZmGASA17
MAKPPLQTATIILLVLLVAASCLHTVDAAALGFSWGKCCVRCARTTTRRAREACMSSCGL
KCEFCKCGPAMPPRDIHDCPCYRNMLTASPKKRPKYP*
>ZmGASA18
MASRNKAAALLLCFLFLAAVAASAAEMIAGSGIGDGEGEELDKGGGGGGGHHKHEGYK
NK

DGKGNLKPSQCGGECRRRCSKTHHKKPCLFFCNKCCAKCLCVPPGTYGNKETCPCYNNW
KTKKGGPKCP*

Tobacco GASA proteins

>NtGASA1
MKSNPFKLIKYFMKSNFIDLHYFIHMKILNFSCYTSNAISEAAYSYPKIDCGGACKARCRLSS
RPRLCKRACGTCCARCNCVPPGTSG>NtETCPCYANMTTHGNRRKCP

>NtGASA2
MAISKTLFVSLVLSLLLLDQVQSIQTDQVTSNAISEAAYSYPKIDCGGACKARCRLSSRPRLC
KRACGTCCARCNCVPPGTSG>NtETCPCYANMTTHGNRRKCP

>NtGASA3
MAISKLILVAMVFFSLLVLHLVEADNQLVV>NtDATESFYTPKLDCGAACEARCRLASRQKIC
KRACGTCCGRCNCVPPGTSGNQELCPCYFAMTTHGGKRKCP

>NtGASA4
MAISKLILVAMVFFSLLVVHLVEADNQVVVNKDATKSSYTPKLDCGAACEARCRLASRQKI
CKRACGTCCARCNCAPPGTSGNLELCPCYFAMTTHGGKRKCP

>NtGASA5
MDISKSKALIVSLIFLLFLLSAAENHELSSSNNIDAISKALKARPNYTINCGKECTRRCKLASR
QKMCMRACGTCCARCNCVPPGTSGNENICPCYSTMTTHGNRRKCP

>NtGASA6
MPIKRPALPHFNHIILNPQENMKFFTLVFIAILLIQVFTEAVSINNAEDTAAQIEKAGNDGAL
FKKSHHHPIRKINCGYACARRCRKSSRKNVCKRACKSCCARCHCVPRGTYGNKEACPCYA
RLKTHGNRPKCP

>NtGASA7
MKIFTLVFIAILLIQVFAEAVSFNNAEDTAAQIEKAGNEGTLFKKIHHQPIRKINCGHACARR
CRKSSRKNVCKRACKSCCARCHCVPPGTYGNKEACPCYARLKTHGNRPKCP

>NtGASA8
MAFQKAFAALLIASLVLVHFTHALQQGNNSKPPAPSPQAPKPLGNITFTTYIFPPRLIYNHITI
ILNSLTLNSEFDYVADCTGACEYRCSESSRPNLCNRACGSCCRTCHCVPPGTSGNYEACPCY
FNLTTHNDTRKCP

>NtGASA9
MAMAIRLVFVMALLLLFLGVKAEVSLTDPKVEEDKSQHFGLSQAFRVFTRGANRRLVQGV
VLKLVKYLNNGDLAVAPAPAPHPSQLDCGGLCKYRCSLHSRPKVCIRACGTCCLRCKCVPP
GTFGNREMCGKCYTEMTTHGNKTKCP

>NtGASA10
ARQHFDASFLAKPPVGPTTCPVPTAQCSSACDQRCSATSHKNNCLMFCNMCCNWCQCVP
PGTFGQKECCSCYNDWKTEQGTPKCP

>NtGASA11
MAHSSLFECSSACDQRCSATSHKNNCLMFCNICCNWCQCVPPGTFGQKECCSCYNDWKT
EQGTPKCP

>NtGASA12
MAYNARLLFLSMFLVLITFSNVVEGYKKLRPEDCEPKCKYRCSATSHKKPCLFFCKKCCAKC
LCVPPGTHGNKETCPCYNNWKTKEGGPKCP

>NtGASA13
MEKMPCLMLLPLLIIMLLLVGTHAKITESPAPQPQPP>NtFPMNGTTPGSLHPQECLPRCTYR

CSKTQYKKPCMFFCQKCCAKCFCVPPGTYGNKQFCPCYNNWKTKRGGPKCP
>NtGASA14
MKLCFATLLVVTLVLTSSFIQTTVAGSDFCDSKCKIRCSKAGRQDRCLKYCGICCNECQCVPS
GTYGNKDECPCYRDKKNSKGKPKCP
>NtGASA15
MKLLLATLLLFTLVLTPSFIQTTMAGSSYCDSKCKLRCAKAGVMDRCLKYCGICCEECKCVP
SGTYGNKHECPCYRDKKNNKGKPKCP
>NtGASA16
MKRIFVAFMLVFALLLTSSFLETATAKSVYCARKCKARCSKAGVKDRCVKYCELCCAKCKC
VPTGTYGNKHQCPCYRDMKNFKGKPKCP
>NtGASA17
SCDSKCAVRRGKAGIAKRCLTYCGIYCNKCNCVPSGNYGNKSECPCYRDMLNSKGKSKCP
>NtGASA18
MKLAMITLLIVSLVLTSSFLQSAVAYDDPSSCDSKCAVRCGKAGIKKRCLTYCGICCNKCNC
VPSGNYGNKSECHCYRDMLNSKGKSKCP

Grape GASA proteins
>VvGASA1
MGIRVLLLVTIMLFCIAEASSDDNNIFEHQHNQAVKGSAGRRLLPFLDCGGLCKERCSLHSR
PNVCTRACGTCCVRCKCVPPGTYGNREMCGTCYTEMTTHGNKPKCP
>VvGASA2
MKHLFPTLLLLSLLLHSCFSQPTTDGAGFCGLKCSKRCSQAAVLDRCMKYCGICCQECKCV
PSGTYGNKHECPCYRDKKNSKGKPKCP
>VvGASA3
MPAPMKGFCDSKCGVRCANAGVYDRCVKYCGICCQECKCVPSGTYGNKSECPCYRDKLN
SKGKPKCP
>VvGASA4
MLSLSMMLLLLVQNNATITEAPTPQPQQSTNGFPMHGVTQGSLHPQECAPRCTTRCSKTA
YKKPCMFFCQKCCAKCLCVPPGTYGNKQFCPCYNNWKTKRGGPKCP
>VvGASA5
MDLFIGHLTEFHGNLSSILDLILEREKQVKKTELHRYLSPILNHILERGVNYLQLLTPGLFFSP
CSAMALKILLLLLASYLLVTKRVSANDEEFGVQATYAKAPVPAPVKTPIPAPPVKPPTVTPNP
PAP>VvKPPT>VvPKPPAPPVSPPTVAPKPPAP>VvPKPPTPPANPPTVAPKPPAPPVKPPT>Vv
PKPPSPPVNPPTPKPPASPVKPPTVAPKPPVPPVTPPTTAPMPPVRARLDCIPLCDQRCKAHS
RKNICVRACMTCCDRCKCVPPGTYGNREKCGKCYTDMTTHGNKPKCP
>VvGASA6
MAKVFALFLLALLAISMLHTTVLASHGHGGHHYDQKNYGPGSLKSFQCPSQCSRRCGKTQ
YHKPCMFFCQKCCKKCLCVPPGYYGNKAVCPCYNNWKTKEGGPKCP
>VvGASA7
MAKLVPLLLLALFSISMVATKVMAKEAQYHLDSGSYGPGSLKSNQCPSQCTRRCSKTQYHK
PCMFFCQKCCAKCLCVPPGYYGNKAVCPCYNNWKTKEGGPKCP
>VvGASA8
MATLRLLLVFVALLVFLAQISSDFNIEGEEMPFVSQ>VvVRGGNRRLMQDIDCGGLCKDRCS
LHSRPNVCVRACGTCCVRCKCVPPGTSGNRELCGKCYTDMTTHGNKTKCP

>VvGASA9
MKPLLATFLLVFLVLSSSFVQNAMAGSSFCDSKCAARCSKAGMKDRCLKYCGICCEECKCV
PSGTYGNKHECPCYKDKKNSKGQPKCP
>VvGASA10
MAISKTLIASLLISLLVYQITEAATTSGDGASSPTEKMDCGGACSARCRLSSRPNLCNRACGT
CCARCNCVPPGTSGNQEICPCYANMTTRGNERKCP
>VvGASA11
MKLFSVFIISILLLQAFAEASLVISNAEHSLTSVDESRDEVALHKKSHPRKINCSYACSRRCRK
ASRKNVCSRACKTCCKRCHCVPPGTYGNKNMCPCYASLKTHGHKPKCP
>VvGASA12
MQVLFNSIKLYKLSDCKSKCAYRCSKAGWHKLCLRACNTCCERCNCVPPGTAGNEDVCP
CYAKMTTHGGRHKCP
>VvGASA13
MTYIYFLVSCKYRCSDTQYRNACLEFCNLCCKKCLCVPSGTYGHKEECPCYNNWKTKEGG
PKCP
>VvGASA14
MWVLGCKFLTYISKKKVSKMAQSSNLQSIFLLLLVAFMLLVDVSIVKCPKACNYRCSDTQYL
NACLEFCNLCCQKCLCVPSGTYGHKEECPCYNNWKTKEGGPKCP

Rice GASA proteins
>OsGASA1
MKTRRAALLMLLLLVVVAAASWPQPCDAASGFCGSKCAVRCGRGRGRGSGCLRSCGLCC
EECNCVPTGSGSTRDECPCYRDMLTAGPRKRPKCP
>OsGASA2
MKLNTTTTLALLLLLLLASSSLQVSMAGSDFCDGKCKVRCSKASRHDDCLKYCGVCCASC
NCVPSGTAGNKDECPCYRDMTTGHGARKRPKCP
>OsGASA3
MAPGKLAVFALLASLLLLNTIKAADYPPAPPLGPPPHKIVDPGKDCVGACDARCSEHSHK
KRCSRSCLTCCSACRCVPAGTAGNRETCGRCYTDWVSHNNMTKCP
>OsGASA4
MEGVGVGVRIRALLCCIAMAAMLLSSYQQGQAEASYMPWPPATPPPPAAAAANSTSTAA
ANNSSSSSSTTAPPQQPTAFPMYGVTPGSLRPQECGGRCAYRCSATAYRKPCMFFCQKCCAS
CLCVLPGTYGNKQSCPCYNDWKTKRGGPKCP
>OsGASA5
MASMAKSLLCISLVAILLLVETTAPHGQAYAIDCGAKCGYRCSKSGRPKMCLRACGTCCQR
CGCVPPGTSGNENVCPCYANMTTHNGRHKCP
>OsGASA6
MASSTKIPFLLLAVLLLLSIAFPSEVMAGGRGRGGGGGGGVAGGGNLRPWECSPKCAGRCS
NTQYKKACLTFCNKCCAKCLCVPPGTYGNKGACPCYNNWKTKEGGPKCP
>OsGASA7
MASSGSKSKTNNGFFVPSPATAMAPCFLLLLIFFFLHVDASAAAASSSHPQLQVQQMQVK
RARSLLQAPKIDCQGTCSGRCANNWKKEMCNKMCNVCCNRCNCVPPGSGQDTRHLCPC
YDTMVNPHNGKLKCP
>OsGASA8

MRVPPLRATTALLATLLVAASFQDLTVAADGGGGVVPVPDSVCDAKCQKRCSLKVAGRCM
GLCKMCCHDCGGCVPSGPYASKDECPCYRDMVSPKSRRPKCP
>OsGASA9
MALAGRLLVLFAIALLAISIAEHKALAKGSTSEHDDNVYQVSKGGQGSLKSYQCSPQCAYR
CSQTQYKKPCLFFCNKCCNACLCVPSGLYGNKGECPCYNNWKTKRGGPKCP
>OsGASA10
MDPASRSLSIIFFLVAVTFVVEVSGQKNEAVYHLFGGEGSLTKNECPGKCSYRCSATSHTTVC
MTYCNYCCERCLCVPSGTY
