# Supplementary material for: Genome-Wide Identification and Characterization of Gibberellic Acid-Stimulated Arabidopsis Gene Family in Pineapple (Ananas comosus)
Source: Int J Mol Sci. 2023 Dec 2;24(23):17063. doi: 10.3390/ijms242317063 (PMC10706908; doi:10.3390/ijms242317063)
Supplement: Supplementary file 1 [file ijms-24-17063-s001.zip › Table S3.pdf]

Table S3: Co-linearity of pineapple with *Arabidopsis thaliana*, tobacco, rice, grape, and maize.

One-to-one orthologous relationships between the GASA gene members in pineapple and Arabidopsis						
AcGASAs Gene Name	AcGASAs Gene ID	AtGASAs Gene ID	Ka	Ks	Ka_Ks	Selection Pressure
AcGASA4	Aco001116.1.v3	AT1G74670.1	0.293513573	NaN	NaN	NaN
AcGASA9	Aco004893.1.v3	AT2G14900.1	0.404990881	NaN	NaN	NaN
AcGASA1	Aco012370.1.v3	AT2G14900.1	0.377971076	NaN	NaN	NaN

One-to-one orthologous relationships between the GASA gene members in pineapple and tobacco						
AcGASAs Gene Name	AcGASAs Gene ID	AtGASAs Gene ID	Ka	Ks	Ka_Ks	Selection Pressure
AcGASA1	Aco012370.1.v3	Nitab4.5_0000604g0040.1	0.310333	NaN	NaN	NaN

One-to-one orthologous relationships between the GASA gene members in pineapple and Rice						
AcGASAs Gene Name	AcGASAs Gene ID	AtGASAs Gene ID	Ka	Ks	Ka_Ks	Selection Pressure
AcGASA4	Aco001116.1.v3	transcript:Os09t0414900-01	0.225606	2.204455	0.102341	Purifying selection
AcGASA7	Aco000979.1.v3	transcript:Os04t0465300-01	0.221825	1.099022	0.201838	Purifying selection
AcGASA9	Aco004893.1.v3	transcript:Os03t0760800-01	0.253144	0.524778	0.482384	Purifying selection
AcGASA9	Aco004893.1.v3	transcript:Os03t0607200-01	0.458561	0.579745	0.79097	Purifying selection
AcGASA2	Aco011225.1.v3	transcript:Os05t0432200-00	0.263677	0.873978	0.301697	Purifying selection
AcGASA10	Aco008536.1.v3	transcript:Os03t0760800-01	0.175211	0.320114	0.54734	Purifying selection
AcGASA5	Aco001067.1.v3	transcript:Os06t0266800-02	0.308747	0.900597	0.342824	Purifying selection
AcGASA1	Aco012370.1.v3	transcript:Os03t0607200-01	0.448303	0.73399	0.610775	Purifying selection
AcGASA1	Aco012370.1.v3	transcript:Os07t0592000-01	0.41438	0.497029	0.833714	Purifying selection

One-to-one orthologous relationships between the GASA gene members in pineapple and Grape

AcGASAs Gene Name	AcGASAs Gene ID	AtGASAs Gene ID	Ka	Ks	Ka_Ks	Selection Pressure
AcGASA11	Aco000098.1.v3	transcript:Vitvi18g00691_t001	0.399228	1.007563	0.396232	Purifying selection
AcGASA11	Aco000098.1.v3	transcript:Vitvi03g00593_t001	0.370613	1.686225	0.219789	Purifying selection
AcGASA4	Aco001116.1.v3	transcript:Vitvi14g03084_t001	0.22285	1.44002	0.154755	Purifying selection
AcGASA4	Aco001116.1.v3	transcript:Vitvi17g00601_t001	0.203233	2.580314	0.078763	Purifying selection
AcGASA9	Aco004893.1.v3	transcript:Vitvi18g00691_t001	0.307553	1.093605	0.281228	Purifying selection
AcGASA9	Aco004893.1.v3	transcript:Vitvi03g00593_t001	0.349635	1.224639	0.285501	Purifying selection
AcGASA9	Aco004893.1.v3	transcript:Vitvi07g01638_t001	0.330929	1.30681	0.253235	Purifying selection
AcGASA6	Aco000980.1.v3	transcript:Vitvi01g00821_t001	0.1533	1.348021	0.113723	Purifying selection
AcGASA6	Aco000980.1.v3	transcript:Vitvi14g01819_t001	0.268448	NaN	NaN	NaN
AcGASA6	Aco000980.1.v3	transcript:Vitvi17g00750_t001	0.144844	0.867075	0.167049	Purifying selection
AcGASA10	Aco008536.1.v3	transcript:Vitvi18g00691_t001	0.246023	1.237165	0.19886	Purifying selection
AcGASA10	Aco008536.1.v3	transcript:Vitvi03g00593_t001	0.303005	1.265189	0.239494	Purifying selection
AcGASA10	Aco008536.1.v3	transcript:Vitvi07g01638_t001	0.30053	1.435237	0.209394	Purifying selection
AcGASA8	Aco002373.1.v3	transcript:Vitvi08g01686_t001	0.252713	NaN	NaN	NaN
AcGASA13	Aco015815.1.v3	transcript:Vitvi07g01086_t001	0.396276	NaN	NaN	NaN
AcGASA1	Aco012370.1.v3	transcript:Vitvi18g00691_t001	0.284223	1.443821	0.196855	Purifying selection
AcGASA1	Aco012370.1.v3	transcript:Vitvi03g00593_t001	0.332918	1.224521	0.271876	Purifying selection
AcGASA1	Aco012370.1.v3	transcript:Vitvi07g01638_t001	0.320309	1.687821	0.189777	Purifying selection

One-to-one orthologous relationships between the GASA gene members in pineapple and Maize						
AcGASAs Gene Name	AcGASAs Gene ID	AtGASAs Gene ID	Ka	Ks	Ka_Ks	Selection Pressure
AcGASA15	Aco010336	Zm00001eb066440_P001	0.269409	0.836017	0.322253	Purifying selection
AcGASA9	Aco004893	Zm00001eb050180_P001	0.498117	0.731897	0.680584	Purifying selection

AcGASA9	Aco004893	Zm00001eb214350_P001	0.313489	0.454405	0.689888	Purifying selection
AcGASA2	Aco011225	Zm00001eb348690_P001	0.325702	1.346662	0.241859	Purifying selection
AcGASA10	Aco008536	Zm00001eb214350_P001	0.291238	0.484199	0.601486	Purifying selection
