# Supplementary material for: Genome-Wide Identification and Characterization of Gibberellic Acid-Stimulated Arabidopsis Gene Family in Pineapple (Ananas comosus)
Source: Int J Mol Sci. 2023 Dec 2;24(23):17063. doi: 10.3390/ijms242317063 (PMC10706908; doi:10.3390/ijms242317063)
Supplement: Supplementary file 1 [file ijms-24-17063-s001.zip › Table S4.pdf]

Table S4: Non-redundant AcGASAS gene lDs associated with the syntenic relationships among pineapple and the other 5 species

**Ananas comosus-Arabidopsis thaliana**	**Ananas comosus-Oryza sativa**	**Ananas comosus-Vitis vinifera**	**Ananas comosus-Zea mays**	**Ananas comosus-Nicotiana tabacum**
AcGASA2	AcGASA2	AcGASA1	AcGASA3	AcGASA15
AcGASA6	AcGASA5	AcGASA2	AcGASA6	
AcGASA15	AcGASA6	AcGASA6	AcGASA7	
	AcGASA7	AcGASA9	AcGASA10	
	AcGASA10	AcGASA10		
	AcGASA14	AcGASA11		
	AcGASA15	AcGASA12		
		AcGASA15		
