# Supplementary material for: Genome-Wide Identification and Characterization of Gibberellic Acid-Stimulated Arabidopsis Gene Family in Pineapple (Ananas comosus)
Source: Int J Mol Sci. 2023 Dec 2;24(23):17063. doi: 10.3390/ijms242317063 (PMC10706908; doi:10.3390/ijms242317063)
Supplement: Supplementary file 1 [file ijms-24-17063-s001.zip › Table S5.pdf]

Table S5: Ka/Ks ratio for the pineapple *AcGASA* gene.

Gene pair name	Ka_Ks
AcGASA1-AcGASA6	0.643300335
AcGASA9-AcGASA10	0.365147124
AcGASA1-AcGASA9	0.35433996
AcGASA2-AcGASA12	0.351344723
AcGASA9-AcGASA10	0.365147124
AcGASA1-AcGASA10	0.542003055
AcGASA2-AcGASA12	0.351344723
