# Supplementary figures and images for: Genome-Wide Identification and Characterization of Gibberellic Acid-Stimulated Arabidopsis Gene Family in Pineapple (Ananas comosus)
Source: Int J Mol Sci. 2023 Dec 2;24(23):17063. doi: 10.3390/ijms242317063 (PMC10706908; doi:10.3390/ijms242317063)

Figure S1: Featured motifs of AcGASA proteins.

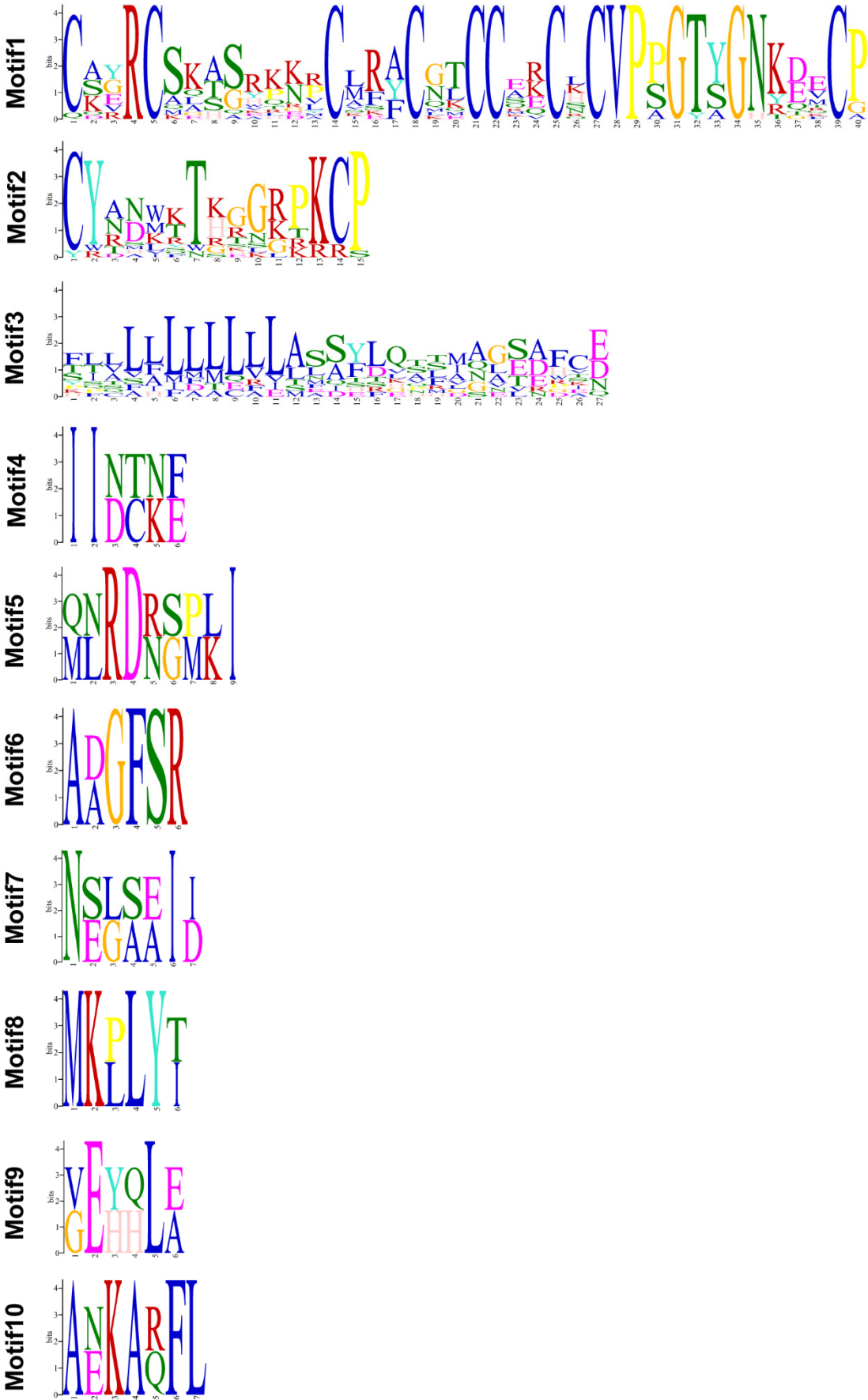

Supplement: Supplementary file 1 [file ijms-24-17063-s001.zip › Figure S1.pdf]
